# Prevalence of dyslipidemia and associated risk factors among newly diagnosed Type-2 Diabetes Mellitus (T2DM) patients in Kushtia, Bangladesh

**DOI:** 10.1371/journal.pgph.0000003

**Published:** 2021-12-07

**Authors:** Md. Saad Ahmmed, Suvasish Das Shuvo, Dipak Kumar Paul, M. R. Karim, Md. Kamruzzaman, Niaz Mahmud, Md. Jannatul Ferdaus, Md. Toufiq Elahi

**Affiliations:** 1 Bangladesh Institute of Research and Training on Applied Nutrition (BIRTAN), Jhenaidah, Bangladesh; 2 Department of Nutrition and Food Technology, Jashore University of Science and Technology, Jashore, Bangladesh; 3 Department of Applied Nutrition and Food Technology, Islamic University, Kushtia, Bangladesh; 4 Food and Nutritional Sciences Program, North Carolina Agricultural and Technical State University, Greensboro, NC, United States of America; African Population and Health Research Center, KENYA

## Abstract

Dyslipidemia is considered a significant modifiable risk factor for type-2 diabetes mellitus (T2DM) and has become one of the emerging health problems throughout the world. In Bangladesh, data on dyslipidemia among newly diagnosed T2DM patients are comparatively inadequate. This study aimed to evaluate the prevalence of dyslipidemia and its associated risk factors in newly diagnosed T2DM patients. This cross-sectional study was conducted by a well-structured questionnaire from 132 newly diagnosed type-2 diabetic patients attending the Mujibur Rahman Memorial Diabetic Hospital in Kushtia, Bangladesh. Data regarding socio-demographic, anthropometric, fasting blood glucose, total cholesterol (TC), triglyceride (TG), high-density lipoprotein (HDL), and low-density lipoprotein (LDL) were collected from all the respondents. The association between dyslipidemia and its associated factors was analyzed using the multivariate logit regression model. The findings suggest that the prevalence rate of dyslipidemia was 75.7% in female and 72.6% in male T2DM patients. The odds of having dyslipidemia were 1.74 (95% Cl: 1.58–1.87) times significantly higher in female (p<0.001). The other factors associated with dyslipidemia encompassed age between 30–39 years (OR: 2.32, 95% CI: 1.97–2.69), obesity (OR: 2.63, 95% CI: 2.27–2.90), waist circumferences of male ≥90 and female ≥80 (OR: 1.65, 95% CI: 1.59–1.89), hypertensive patients (OR: 1.51, 95% CI: 1.45–1.74), physically inactive (OR: 3.25, 95% CI: 1.84–4.68), and current smoker or tobacco user (OR: 1.93, 95% CI: 1.85–2.13). This study concluded that the high prevalence of dyslipidemia was found among newly diagnosed type-2 diabetes patients and associated with gender, age, BMI, waist circumference, poor physical activity, and smoking, or tobacco use. This result will support increase awareness of dyslipidemia and its associated risk factors among type-2 diabetes patients.

## 1. Introduction

Diabetes mellitus (DM) is a chronic disease condition associated with hyperglycemia resulting from an imbalance in insulin secretion and insulin action or cooperation of [1, 2]. Estimation studies have confirmed that the incidence of DM in people worldwide in 2019 was 463 million and is anticipated to reach 642 million by 2040, becoming one of the biggest global public health problems [3]. Of all cases of diabetes, more than 90% are detected as type-2 diabetes mellitus (T2DM) around the world [3]. Several metabolic syndromes (MetS) including dyslipidemia, hyperglycemia, and hypertension become a channel to exacerbating cardiovascular disease (CVD) risk factors in T2DM patients [4, 5]. Metabolic syndrome (MetS) comprises several situations: elevated glucose and blood pressure level and abnormal cholesterol or triglyceride levels [6]. In particular, the MetS is recognized by a condition whereby the body’s cells cannot take up glucose from the blood [7].

In recent years, there has been much attention given to understanding the MetS, which is found to be responsible for predicting T2DM [7, 8]. The most common significant component of MetS that occurs in T2DM is dyslipidemia, characterized by hypertriglyceridemia, reduced high-density lipoproteins (HDL) cholesterol levels, and an increased concentration of small dense low-density lipoproteins (LDL) particles [7, 8]. Dyslipidemia is a common phenomenon in type-2 diabetic patients characterized by an abnormal lipid profile because insulin resistance or deficiency affects key enzymes and pathways in lipid metabolism [9, 10]. Globally, the prevalence of dyslipidemia is gradually increasing (≥80% to 90%) in developing countries like Ethiopia [11], Kenya [12], Sri Lanka [13], India [14], and Bangladesh [15, 16]. Cardiovascular risk is significantly increased among patients with diabetes by the presence of dyslipidemia [7]. Studies show that approximately 70–80% of people having diabetes will die of cardiovascular disease [17, 18]. Over the years, to elucidate the involvement and impact, researchers worldwide have been investigating the association between T2DM and dyslipidemia.

Globally, several community-based studies found that blood pressure, fasting blood glucose, BMI, age, poor physical activity, dietary habits, smoking/tobacco use, and other lifestyle changes have contributed as the major risk factors for increasing the prevalence of dyslipidemia among T2DM patients [11, 16, 19–21]. For example, in some Asian countries, several studies observed a rising trend in the prevalence of dyslipidemia and other metabolic syndromes in T2DM patients [13, 22–25]. However, very few studies are conducted in Bangladesh to assess the prevalence of dyslipidemia in T2DM patients [15, 16]. Insofar as the author’s knowledge, factors associated with dyslipidemia among T2DM patients in Bangladesh are still relatively scarce. In the wake of the rising incidence of diabetes in Bangladesh, there is an urgency to initiate investigation towards evaluating the prevalence and associated risk factors of dyslipidemia among newly diagnosed T2DM patients. So this study will help health professionals and health policymakers to provide better management, appropriate program intervention, and treatment approaches to dyslipidemia. Therefore, to understand the magnitude of dyslipidemia in newly diagnosed T2DM patients and fill the gap of knowledge, this study aimed to evaluate the prevalence of dyslipidemia and to find out the associated risk factors among newly diagnosed T2DM patients.

## 2. Materials and methods

### 2.1 Study design and settings

A total of 132 subjects with a new diagnosis of T2DM were included in this cross-sectional study. The study was conducted at the Mujibur Rahman Memorial Diabetic Hospital, Hospital Road, at Kushtia District, Bangladesh, from March to November 2017. A purposive sampling technique was deployed for various reasons. Firstly, we need to collect newly diagnosed T2DM patients. Secondly, this study needs to exclude previously detected diabetes patients and pregnant women with diabetes. Finally, purposive sampling was used to recruit participants who can provide in-depth and detailed information about the phenomenon under investigation.

### 2.2 Data collection instrument and techniques

A self-administrated and semi-structured questionnaire was used to gather the data through a face-to-face interview. The questionnaire was structured in three sections included socio-demographic characteristics, blood specimen collection, and anthropometric assessment. Two trained interviewers and a medical technologist were employed to collect the data. The survey questionnaire was formatted in the English language then converted to the Bengali language for easy understanding. After collecting information, the questionnaire was then translated back into the English version. Before the pilot study, the questionnaire was pre-tested two times at the same study place. The pre-tests outcomes were satisfactory and expected that’s why no modification was needed in the final questionnaire. However, the pre-tested data were not used in the final data analysis. Prior to the final data collection, the aim of the study was explained in detail to each study participant. A written agreement was obtained from each study participant, and the study participants were given the full right to withdraw from the study at any time. Data were collected from the participants at the selected diabetic hospital, where each questionnaire took an average of 20 minutes to be administrated.

### 2.3 Exclusion and inclusion criteria

We exclude those T2DM patients who were previously detected as diabetic positive. Pregnant women were excluded though they were first seen as diabetic positive. Those T2DM patients below 30 years and above 70 years were excluded. Persons who were firstly detected as diabetic positive but had been taking drugs to treat hypertension were also excluded. Patients achieving the ADA criteria for diagnosis of type 2 DM: HbA1c > 6.5%, or FBS > 126 mg/dl, or PPBS > 200 mg/dl15 and aged ≥30 years, who attended/admitted in the medicine OPD/IPD with symptoms and signs of diabetes mellitus for the first time, were included as study subject [26, 27]. Dyslipidemia was diagnosed if patients had one or more lipid profile parameters outside the target values recommended by the American Diabetes Association (ADA) [28, 29].

### 2.4 Specimen collection, blood sampling, and biochemical analysis

Fasting overnight venous blood samples (nearly 6ml) were drawn from 132 freshly diagnosed T2DMS patients by a well-trained medical technologist into vacutainer tubes from each diabetic individual. Some standard guidelines were followed to determine the Diagnostic Criteria [30]. The blood was allowed to the left for a while without anticoagulants to allow blood to clot. Then serum sample was obtained by centrifugation at room temperature by Rotina 46 Hettich centrifuge, Japan at 4000 rpm/10 minutes. For each selected subject, overnight fasting blood samples were analyzed for fasting blood glucose (FBG) and lipid profiles, namely total cholesterol (TC), triglyceride (TG), high-density lipoprotein (HDL), and low-density lipoprotein (LDL). Dyslipidemia was assessed according to the United States National Cholesterol Education Program Adult Treatment Panel III (NCEP-ATP-III) model guideline [31]. The Serum glucose, serum cholesterol, serum triglycerides, serum High-Density Lipoprotein (HDL), and Low-Density Lipoprotein (LDL) were determined using the Bio-systems kit (HUMAN GmbH, Wiesbaden, Germany).

### 2.5 Anthropometrics assessment and other variables

Ensuing the standard recommendations of the World Health Organization, trained personnel were recruited to collect the anthropometric measurements. The participants were measured in light clothing and without shoes [29]. Body weight was measured to the nearest 0.1 kg and height to the nearest 0.1 cm. Body mass index (BMI) was then calculated by standard BMI formula [30]. Waist circumference was measured at the midpoint between the lower rib and iliac crest with a flexible steel tape measure to the nearest centimeter while the subjects were in the standing position at the end of gentle expiration [32, 33]. Blood pressure was measured using a sphygmomanometer by the auscultator method [34]. The classification guidelines were used during the study [34]. Gender, age, physical activity, and smoking or tobacco use were recorded with a questionnaire.

### 2.6 Statistical analysis

Descriptive statistics and frequency tables of the baseline characteristics were calculated to describe the study variables. The data were analyzed using the relevant tests of significance, such as the unpaired t-test and Chi-square test. The analytical frameworks used for data analysis were embedded in STATA. Multivariate logit regression analyses were performed using odds ratios (ORs) and 95% CIs to evaluate the independent factors (gender, age, BMI, waist circumference, blood pressure type, physical activity, and smoking/tobacco use) associated with the risk of dyslipidemia. P-value ≤0.05 was considered as the level of significance.

### 2.7 Ethical statement

This study’s protocol was approved by the Research Ethical Committee (REC) of the Department of Applied Nutrition and Food Technology, Islamic University, Kushtia, 7003, Bangladesh. Ethical clearance was also obtained from the ethical committee of the Hospital.

## 3. Results

### 3.1 Prevalence of dyslipidemia among newly diagnosed T2DM patients

Newly diagnosed type-2 diabetic patients’ characteristics and dyslipidemia prevalence are stated in Table 1. Among the total respondents, the majority was female (53%). The prevalence of dyslipidemia in males and females was 72.6% and 75.7%. In terms of age categories, this table also presents that the prevalence of dyslipidemia was higher among 40–49 years aged (76.6%) and 60–70 years aged (74.1%) respondents. Nearly, 34.9% and 14.4% were overweight and obese, whereas the prevalence of dyslipidemia was also higher among overweight (72.4%) and obese (74.9%) patients, respectively. The prevalence of dyslipidemia in hypertensive patients was 73.5%. Moreover, 74.8% and 73.5% currently smoker/tobacco users and past smoker/tobacco user patients were dyslipidemia.

**Table 1:** T2DM respondents characteristics and dyslipidemia prevalence.

Characteristics	Total n (%)	Dyslipidemia n (%)
**Gender**		
Male	62 (47.0)	45 (72.6)
Female	70 (53.0)	53 (75.7)
**Age (Years)**		
30–39	35 (26.5)	22 (62.4)
40–49	43 (32.6)	33 (76.6)
50–59	35 (26.5)	25 (71.4)
60–70	19 (14.4)	14 (74.1)
**Body Mass Index (BMI)**		
Underweight	4 (3.1)	3 (75.0)
Normal	63 (47.7)	44 (69.9)
Overweight	46 (34.8)	33 (72.4)
Obese	19 (14.4)	14 (74.9)
**Waist circumference (cm)**		
Male < 90, Female < 80	67 (50.7)	43 (72.3)
Male ≥90, Female ≥80	65 (49.3)	49 (74.9)
**Blood pressure types**		
Normotensive	54 (40.9)	37 (67.8)
Hypertensive	78 (59.1)	57 (73.5)
**Physical exercise**		
No	83 (63.1)	66 (79.2)
Yes	49 (36.9)	28 (57.2)
**Smoking/Tobacco use**		
No	43 (32.5)	27 (63.7)
Yes	59 (44.6)	44 (74.8)
Ex-smoker/ Tobacco user	30 (22.9)	22 (73.5)

Table 2 illustrates the significance of gender in different clinical health statuses. Respondents’ gender was found significant (p<0.05 and p<0.01) to the BMI and blood pressure. The majority of female respondents were found overweight and obese than their male counterparts, and they were 38.6% and 20% for female respondents and 30.6% and 8.1% for male respondents, respectively. However, the gender was not found significant to the respondents’ blood LDL, TG, HDL, and TC levels. Most of the respondents (59.1%) had high blood pressure, where an equal number of people had desirable and risk levels of LDL in the blood. However, most of the study participants had a risk level of blood TG, HDL, and TC; 57.6%, 74.2%, and 52.3%, respectively.

**Table 2:** Relationship between different clinical status and gender of the respondents.

Variables	Male n (%)	Female n (%)	Total n (%)	Chi^2^—value^¥^	P—value^†^
**Age category**					
30–39 years	14 (22.6)	21 (30)	35 (26.5)	2.27	0.518
40–49 years	24 (38.7)	19 (27.1)	43 (32.6)		
50–59 years	15 (24.2)	20 (28.6)	35 (26.5)		
60–70 years	9 (14.5)	10 (14.3)	19 (14.4)		
**BMI category**					
Underweight	1 (1.6)	3 (4.3)	4 (3.1)	8.12	**0.044**
Normal weight	37 (59.7)	26 (37.1)	63 (47.7)		
Overweight	19 (30.6)	27 (38.6)	46 (34.8)		
Obese	5 (8.1)	14 (20.0)	19 (14.4)		
**Waist circumference (cm)**					
Male < 90, Female < 80	38 (61.3)	29 (41.4)	**67 (50.7)**	3.65	0.735
Male ≥90, Female ≥80	24 (38.7)	41 (58.6)	**65 (49.3)**		
**Blood pressure types**					
Normotensive	33 (53.2)	21 (30.0)	54 (40.9)	7.33	**0.007**
Hypertensive	29 (46.8)	49 (70.0)	78 (59.1)		
**Smoking/Tobacco use**					
No	12 (19.4)	31 (44.3)	43 (32.5)	9.45	0.03
Yes	35 (56.4)	24 (34.3)	59 (44.6)		
Ex-smoker/ Tobacco user	15 (24.2)	15 (21.4)	30 (22.9)		
**Physical exercise**					
No	32 (51.7)	51 (72.6)	83 (63.1)	15.62	0.001
Yes	30 (48.3)	19 (27.4)	49 (36.9)		
**Level of LDL**					
Desirable (<130mg/dL)	35 (56.5)	31 (44.3)	66 (50.0)	1.94	0.111
Risk (>130mg/ dL)	27 (43.5)	39 (55.7)	66 (50.0)		
**Level of TG**					
Desirable (<150 mg/dL)	29 (46.8)	27 (38.6)	56 (42.4)	0.91	0.341
Risk (>150 mg/dL)	33 (53.2)	43 (61.4)	76 (57.6)		
**Level of HDL**					
Desirable (>40 mg/dL)	17 (27.4)	17 (24.3)	34 (25.8)	0.16	0.681
Risk (<40 mg/dL)	45 (72.6)	53 (75.7)	98 (74.2)		
**Level of TC**					
Normal (<200 mg/dL)	27 (43.5)	36 (51.4)	63 (47.7)	0.81	0.366
Abnormal (>200 mg/dL)	35 (56.5)	34 (48.6)	69 (52.3)		

**Note:** BMI = Body Mass Index; BP = Blood Pressure; LDL = Low Density Lipoprotein; TG = Triglycerides; HDL = High Density Lipoprotein; TC = Total Cholesterol

^**¥**^Values were obtained from crosstabulation

^**†**^Values were obtained from one-way ANOVA.

The significance of the blood pressure types on the respondents’ different clinical health statuses is demonstrated in Table 3. The blood pressure type was found significant (p ≤ 0.05 and p≤ 0.01) to all clinical health status of the studied population except the LDL level in the blood. Overall, 40.9% and 59.1% of respondents had normotensive and hypertensive blood pressure, while 70% of the hypertensive and 53.2% of the normotensive respondents were female and male, respectively. Majority of the hypertensive respondents were also in the 50–59 years age group. In the BMI category, 46.2% and 12.8% of the hypertensive respondents were overweight and obese. The TG level in blood had significance (p<0.01) to blood pressure type and 71.8% of the hypertensive respondents had a risk level (>150 mg/dL) of blood TG. Around 80% of the hypertensive respondents and 64.8% of the normotensive respondents had a risk level (<40 mg/dL) of blood HDL. Moreover, blood pressure was identified as a more significant (p<0.01) factor to the level of TC in blood, where 61.5% of the hypertensive people had abnormal levels (>200 mg/dL) of blood TC.

**Table 3:** Relationship between different clinical status and blood pressure types of the respondents.

Variables	Normotensive n (%)	Hypertensive n (%)	Total n (%)	Chi^2^-value^¥^	P- value^†^
**Gender**					
Male	33 (53.2)	21 (30.0)	54 (40.9)	7.33	0.007
Female	29 (46.8)	49 (70.0)	78 (59.1)		
**Age category**					
30–39 years	20 (37)	15 (19.2)	35 (26.5)	17.57	0.001
40–49 years	23 (42.6)	20 (25.6)	43 (32.6)		
50–59 years	5 (9.3)	30 (38.5)	35 (26.5)		
60–70 years	6 (11.1)	13 (16.7)	19 (14.4)		
**BMI category**					
Underweight	2 (3.7)	2 (2.5)	4 (3.1)	8.12	0.044
Normal weight	33 (61.1)	30 (38.5)	63 (47.7)		
Overweight	10 (18.5)	36 (46.2)	46 (34.8)		
Obese	9 (16.7)	10 (12.8)	19 (14.4)		
**Waist circumference (cm)**					
Male <90, Female <80	35 (64.8)	32 (41.0)	67 (50.7)	7.64	0.03
Male ≥90, Female ≥80	19 (35.2)	46 (59.0)	65 (49.3)		
**Smoking/Tobacco use**					
No	23 (42.6)	20 (25.7)	43 (32.5)	11.92	0.002
Yes	15 (27.8)	44 (56.4)	59 (44.6)		
Ex-smoker/ Tobacco user	16 (29.6)	14 (17.9)	30 (22.9)		
**Physical exercise**					
No	17 (31.5)	66 (84.6)	83 (40.9)	15.62	0.001
Yes	37 (68.5)	12 (15.4)	49 (59.1)		
**Level of LDL**					
Desirable (<130mg/dL)	32 (59.3)	34 (43.6)	66 (50.0)	3.13	0.055
Risk (>130mg/ dL)	22 (40.7)	44 (56.4)	66 (50.0)		
**Level of TG**					
Desirable (<150 mg/dL)	34 (63.0)	22 (28.2)	56 (42.4)	15.78	0.001
Risk (>150 mg/dL)	20 (37.0)	56 (71.8)	76 (57.6)		
**Level of HDL**					
Desirable (>40 mg/dL)	19 (35.2)	15 (19.2)	34 (25.8)	4.24	0.039
Risk (<40 mg/dL)	35 (64.8)	63 (80.8)	98 (74.2)		
**Level of TC**					
Normal (<200 mg/dL)	33 (61.1)	30 (38.5)	63 (47.7)	6.56	0.010
Abnormal (>200 mg/dL)	21 (38.9)	48 (61.5)	69 (52.3)		

Note

^**¥**^Values were obtained from cross-tabulation

^**†**^Values were obtained from one-way ANOVA.

Table 4 illustrates the significance level of the gender and the blood pressure type on the different variable and their mean value. This table shows that gender was not a significant factor in the mean value of the different variables. Simultaneously, blood pressure had a high significance (p<0.01) level of effect on the respondents’ clinical status. The respondents’ average age in the hypertensive group was around 50 years, and they were overweight (BMI > 24.99). In terms of total cholesterol (TC) and triglycerides (TG) level in the blood, these were found high in hypertensive respondents, and the values were 212.6mg/dL and 229.3mg/dL, respectively. Moreover, systolic and diastolic blood pressures were also found at a high level in hypertensive people, and the values were 138.9mmHg, and 96.1mmHg, respectively.

**Table 4:** Mean±SD and significance level of different variables based on blood pressure and gender of the respondents.

Variables	Gender		t-value^£^	p-value^£^	BP types		t-value^£^	p-value^£^
	Male	Female			Normotensive	Hypertensive		
**Age**	46.5±10.8	46.33±10.5	-0.11	0.91	42.5±10.1	49.1±10.1	-3.71	**0.001**
**BMI**	24.4±3.5	25.82±4.4	1.96	0.05	23.7±4.4	25.1±5.8	-3.57	**0.001**
**FBG Level**	9.4±3.9	9.36±4.1	-0.06	0.94	9.6±4.3	9.2±3.8	0.57	0.566
**A. 75g glu.**	17.5±4.2	17.47±4.0	-0.04	0.96	17.2±4.2	17.7±4.0	-0.67	0.498
**TC (**mg/dL)	204.9±40.9	218.70±75.8	-0.33	0.74	190.4±38.3	212.6±45.3	-2.94	**0.004**
**TG (**mg/dL)	208.1±59.1	218.70±75.8	0.48	0.62	196.9±52.1	229.3±71.0	-2.86	**0.005**
**LDL (**mg/dL)	110.2±31.5	117.17±33.9	1.21	0.22	109.7±35.5	116.8±30.8	-1.22	0.224
**HDL (**mg/dL)	34.7±6.5	36.11±6.8	1.23	1.43	36.5±8.1	34.6±5.4	1.49	0.139
**S. pressure**	131.4±12.7	131.79±13.7	0.18	0.85	121.0±11.4	138.9±8.5	-10.30	**0.001**
**D. pressure**	87.5±9.1	90.07±10.6	1.47	0.14	78.4±5.7	96.1±4.2	-20.35	**0.001**

**Note:** FBS = Fasting Blood Glucose; A. 75g glu. = After having 75g glucose; S. pressure = Systolic-blood pressure; D. pressure = Diastolic-blood pressure

^**£**^values were obtained from independent sample t-taste.

### 3.2 Factors associated with dyslipidemia among newly diagnosed T2DM patients

Multivariate logit regression analyses were used to determine the factors associated with dyslipidemia in T2DM patients are presented in Table 5. The odds of having dyslipidemia was 1.74 times (95% CI: 1.58–1.87) higher in female compared with male counterparts (p < 0.001). The patients aged between 40–49 years had 2.32 times (95% CI: 1.97–2.69) higher odds in comparison with those between 30–39 years (p<0.001). Besides, patients aged 50–59 years and 60–70 years had 1.87 times and 1.71 times increased the risk of having dyslipidemia than patients aged between 30–39 years. The results also shows that patients with a higher BMI were more likely to dyslipidemia disease, where obesity is associated with 2.63 times (95% CI: 2.27–2.90) increased odds than underweight (p<0.001). Male with a waist circumference ≥90 cm and female with a waist circumference ≥80 cm had 1.65 times higher odds of having dyslipidemia (p<0.001). Remarkably, the hypertensive (OR: 2.51, 95% CI: 1.45–3.74) and physically inactive (OR: 3.25, 95% CI: 1.84–4.68) patients were associated with increased odds of having dyslipidemia relative to normotensive and physically inactive patients (p ≤0.001). Moreover, the odds of having dyslipidemia were 1.93 times (95% CI: 1.85–2.13) and 1.72 times (95% CI: 1.65–1.90) higher in current and ex-smoker/tobacco users compared with non-smoker/tobacco users (p<0.04).

**Table 5:** Logit-regression analysis of associated factors with dyslipidemia in T2DM patients.

Variables	Total (%)	Dyslipidemia (%)	Odds ratio (95% CI)	P—value
**Gender**				
Male	47.0	72.6	1	
Female	53.0	75.7	1.74 (1.58–1.87)	<0.001
**Age (years)**				
30–39	26.5	62.4	1	
40–49	32.6	76.6	2.32 (1.97–2.69)	0.003
50–59	26.5	71.4	1.87 (1.75–2.10)	< 0.001
60–70	14.4	74.1	1.71 (1.59–1.84)	< 0.001
**BMI categories**				
Underweight	3.1	61.6	1	
Normal weight	47.7	69.8	1.26 (1.20–1.42)	0.06
Overweight	34.8	72.4	2.08 (1.73–2.23)	< 0.001
Obese	14.4	74.9	2.63 (2.27–2.90)	< 0.001
**Waist circumference (cm)**				
Male < 90, Female < 80	50.7	72.3	1	
Male ≥90, Female ≥80	49.3	74.9	1.65 (1.59–1.89)	< 0.001
**Blood pressure types**				
Normotensive	40.9	67.8	1	
Hypertensive	59.1	73.5	2.51 (1.45–3.74)	< 0.001
**Physical exercise**				
Yes	36.9	57.2	1	
No	63.1	79.2	3.25 (1.84–4.68)	0.001
**Smoking/Tobacco use**				
No	32.5	63.7	1	
Yes	44.6	74.8	1.93 (1.85–2.13)	0.04
Ex-smoker/ Tobacco user	22.9	73.5	1.72 (1.65–1.90)	0.01

## 4. Discussion

The prevalence of dyslipidemia is gradually increasing in developing countries because of economic development, modifiable lifestyle, and physical inactivity [15, 19, 20, 35]. This present study was piloted to assess the prevalence of dyslipidemia and associated risk factors among newly diagnosed type-2 diabetes patients visiting Kushtia diabetic hospital, Bangladesh. In this recent study, the total prevalence rate of dyslipidemia was high in both male (72.6%) and female respondents (75.7%). This finding is also similar to other studies in different countries that agreed with the high prevalence [13, 15, 16, 22]. This study also estimated that the prevalence rate of lipid profiles was also in alarming conditions that were LDL (50%), TG (57.60%), HDL (74.20%), and TC (52.30%), respectively. A recent study in the southern region of Bangladesh showed that the prevalence of LDL, TG, HDL, and TC was at high level [15]. On the contrary, some previous studies in different countries were found that the dyslipidemia prevalence rate was high in studied populations while they had the risk level of serum LDL, TG, HDL, and TC [19, 36, 37]. Additionally, recent studies conducted in Ethiopia, Thailand, and Nepal explained that serum lipid profile was the significant factor for increasing the blood pressure level of diabetes [11, 23, 24]. Expectedly, the lipid profile namely, TG, HDL, and TC level patients, among them the level of TG and TC were found as the more significant (p≤0.01) factors [38].

In contrast with the prevalence of dyslipidemia, this study also evaluated some potential factors that may increase the risk of dyslipidemia among newly diagnosed type-2 diabetic patients. This study found that females had higher odds of being dyslipidemia in type-2 diabetes patients. Similar studies reported that female diabetic patients were significantly associated with dyslipidemia, which could be ascribed to their working hours and work spheres [1, 11, 13]. This study also showed that increased age was positively associated with dyslipidemia. This result is in line with other studies [11, 19, 20, 24, 35, 38]. Though no evidence has yet been identified that age directly impacts serum lipid profiles but inherited genetic characteristics, insulin resistance and degenerative processes might be associated with age [20]. Another study found that increasing age was associated with dyslipidemia in type-2 diabetic patients because of their workload and poor physical activity [14, 39]. The study revealed that obesity was significantly associated with dyslipidemia in T2DM patients (p<0.001). Similar findings also found in other studies, dyslipidemia is more prevalent among T2DM obese patients compared with normal-weight people [15, 24, 40, 41]. Different studies also reported that obesity helps to release a high amount of free fatty acids by lipolysis which leads to developing hypertriglyceridemia. Along with, the liver also increased the production of very-low-density lipoprotein and triglyceride that are the potential contributor to developing CVD and atherosclerosis diseases in T2DM patients [40, 41]. Moreover, researchers also have examined that obesity increase lipid profile and significantly impact on HbA1c level, as a result of insulin inactivity [42, 43].

Additionally, this study confirmed that hypertension was significantly associated with the prevalence of dyslipidemia in diabetic patients which is similar to those reported in other studies [11, 35, 44]. Another important finding of this study showed that physical inactivity was significantly associated with dyslipidemia among T2DM patients. This finding was consistent with other studies from Ethiopia [11], China [21], Thailand [24], and Saudi Arabia [19]. Several recent studies explained that poor physical activity and dietary habits potentially increase the blood glucose level which may lead to dyslipidemia in T2DM patients [19, 45]. From the pieces of evidence, it has been concluded that regular exercise helps to control glycemic and lipid profile in diabetes patients. The results also showed that current smoking was significantly related to an increase in the risk of dyslipidemia prevalence in diabetic patients. This is following similar studies that found an association between dyslipidemia and smoking [19, 24, 25, 46]. Importantly, other studies also investigated that smoking might increase the LDL-cholesterol, TG but decreases the HDL-cholesterol level that promotes dyslipidemia [46–48].

Finally, the findings of this study along with some other studies reported that the prevalence of dyslipidemia is increasing at an alarming rate in our country. Therefore the implementation of comprehensive national public policy is urgently needed. The risk factors of increasing dyslipidemia can be reduced by the implementation of healthy public policy, adequate knowledge regarding controlling factors of diabetes, and healthy lifestyle interventions. The primary health care facilities should execute regular follow-up, monitor, proper advice, and intervention programs to reduce the prevalence of dyslipidemia among type 2 diabetes patients.

However, this study has also some potential limitations. Firstly, this was a single assessment study of blood samples in newly diagnosed T2DM patients to determine the prevalence of dyslipidemia. Secondly, dietary diversity was not assessed to identify the effect of serum lipid. Thirdly, this study might be lead to self-reported bias due to the questionnaire survey. Finally, this cross-sectional design confines to address the causative relationships amid associated risk factors and dyslipidemia prevalence.

## 5. Conclusion

In summary, this study may be concluded that the prevalence of dyslipidemia among newly diagnosed type-2 diabetes was considerably high in both male and female respondents. Associated factors including gender, age, BMI, waist circumferences, BP type, poor physical activity, and smoking or tobacco use increased the risk ratio of dyslipidemia. These findings might be supportive for planning effective policies and raising awareness regarding the better management of lipid profiles to reduce the prevalence of dyslipidemia in diabetic patients. Therefore, this study highlights the emergency need for screening lipid profile, treatment, and implementation of diabetic education knowledge about dyslipidemia among T2DM patients.
